# Expression of inwardly rectifying potassium channels by an inducible adenoviral vector reduced the neuronal hyperexcitability and hyperalgesia produced by chronic compression of the spinal ganglion

**DOI:** 10.1186/1744-8069-6-65

**Published:** 2010-10-06

**Authors:** Chao Ma, Jason Rosenzweig, Pu Zhang, David C Johns, Robert H LaMotte

**Affiliations:** 1 Dept. Anesthesiology, Yale University School of Medicine, New Haven, CT, USA; 2 Dept. Neurosurgery, The Johns Hopkins Hospital, Baltimore, MD, USA; 3 The Wilmer Gene Vector Core, The Johns Hopkins University School of Medicine, Baltimore, MD, USA

## Abstract

**Background:**

A chronic compressed dorsal root ganglion (CCD) in rat produces pain behavior and an enhanced excitability of neurons within the compressed ganglion. Kir2.1 is an inwardly rectifying potassium channel that acts to stabilize the resting potential of certain cell types. We hypothesized that an inducible expression of Kir2.1 channels in CCD neurons might suppress neuronal excitability in the dorsal root ganglion (DRG) and reduce the associated pain behavior.

**Results:**

We delivered, by microinjection into the fourth lumbar (L4) DRG, an adenoviral vector containing a reporter gene encoding the enhanced green fluorescent protein (GFP) and a Kir2.1 channel (AdKir). At the same time the ganglion was compressed by implantation of a rod through the intervertebral foramen (CCD). The *in vivo *expression of the transferred gene was controlled by an ecdysone analog via an ecdysone-inducible promoter in the viral vector. In comparison with the effects of vehicle or a control vector containing only the GFP gene, AdKir significantly reduced the neuronal hyperexcitability after CCD. Electrophysiological recordings, in vivo, from nociceptive and non-nociceptive DRG neurons expressing the virally produced Kir2.1 channels revealed a hyperpolarized resting membrane potential, an increased rheobase, and lack of spontaneous activity. Inducing the Kir2.1 gene at the beginning of CCD surgery partially prevented the development of mechanical hyperalgesia. However, a delayed induction of the Kir2.1 gene (3 days after CCD surgery) produced no significant effect on the pain behavior.

**Conclusions:**

We found that an inducible expression of Kir2.1 channels in chronically compressed DRG neurons can effectively suppress the neuronal excitability and, if induced at the beginning of CCD injury, prevent the development of hyperalgesia. We hypothesize that a higher level of neuronal hyperexcitability in the DRG is required to initiate than to maintain the hyperalgesia and that the hyperexcitability contributing to neuropathic pain is best inhibited as soon as possible after injury.

## Background

The increased excitability of dorsal root ganglion (DRG) neurons associated with an injury of a peripheral nerve or the ganglion may contribute to pain-related behaviors in different animal models of neuropathic pain. After a chronic compression of the DRG (CCD) which produced pain and hyperalgesia in rats, the somata of DRG neurons became hyperexcitable, some with spontaneous activity (SA), both in the intact ganglion and after acute dissociation and placement in culture[1-5].

**Figure 1:**
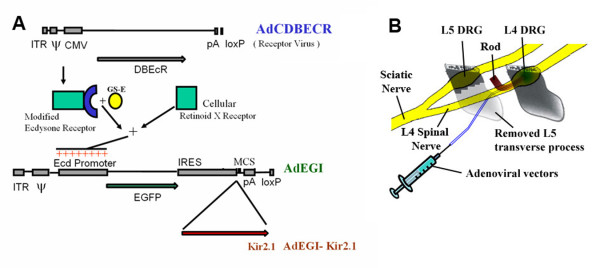
**The structure and method of application of the viral vectors**. A: Schematic representation of the ecdysone-inducible adenovirus vectors and method of application to the DRG. ITR: inverted terminal repeat; ψ: Packaging signal; Ecd promoter: ecdysone-inducible promoter; EGFP: enhanced green fluorescent protein; IRES: internal ribosome entry site; MCS, multiple cloning site; pA, SV40 polyadenylation signal; DBEcR, hybrid ecdysone receptor. AdCDBEcR: receptor virus. AdEGI: the control vector containing only the EGFP gene. AdEGI-Kir2.1: the viral vector containing both the EGFP and the Kir2.1 gene, which encodes an inward-rectifying potassium channel (See main text and Johns et al. 1999 [9] for further details). B: Procedures for sub-epineurial injection of viral vectors into the L4 DRG and rod implantation. Under a dissecting microscope, the L5 transverse process was removed to expose the L4 spinal nerve. A polyethylene tube (tip diameter 100 μm) connected to a microinjection syringe was inserted into the L4 spinal nerve under the epineurium until the tip reached the center of DRG. The viral vectors or vehicle were slowly injected into the sub-epineurial space of DRG using a microinjection pump (5 μL in about 10 min). For CCD surgery, a stainless steel rod was inserted into the intervertebral foramen to compress the L4 DRG.

An adenoviral vector carrying the inwardly rectifying potassium channel, Kir2.1 was shown to reduce the excitability of superior cervical ganglion neurons, in vitro [6]. In the present study, transgenic delivery in vivo was achieved by a sub-epineurial injection of recombinant adenoviral vectors into the DRG of adult rats (Figure 1). The expression of the transferred gene was controlled by an ecdysone analog in vivo via an ecdysone-inducible promoter in the viral vector [7]. By applying adenoviral vectors carrying Kir2.1, we expected to decrease the excitability of DRG neurons, and reduce the pain-related behaviors of the animal after CCD surgery. Some preliminary results of this study have been published in abstract form [8,9].

## Results

### Adenoviral vectors induced mild mechanical hyperalgesia in naïve rats

**Figure 2:**
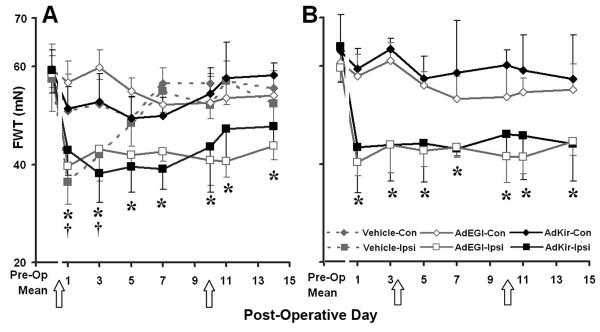
**Behavioral effects of the adenoviral vector in the absence of CCD**. Either the vehicle, the control vector (AdEGI), or the vector containing the Kir2.1 gene (AdKir) was delivered to the L4 DRG via a sub-epineurial injection. The induction agent was then delivered, IP (arrow), either immediately (postoperative day 0) (A) or after a delay of three days (B). In either case, a second induction was delivered on the 10^th ^postoperative day. FWT: Mean threshold force (mN) for foot withdrawal. Injection of vehicle alone produced on the ipsilateral foot, a transient mechanical hyperalgesia (significant decrease in FWT from preoperative values) that recovered within 5 days (A). Ipsilateral (Ipsi) but not contralateral (Con) to the CCD, an injection of vehicle containing either AdEGI or Adkir, produced a mild and long-lasting mechanical hyperalgesia regardless of whether the inducing agent was given immediately or after a delay of 3 days suggesting that the hyperalgesia produced by the presence of virus was not reduced by the expression of Kir. *: P < 0.05 vs. pre-operative mean for the ipsilateral foot of AdEGI group and AdKir groups (A and B); †: P < 0.05 vs. pre-operative mean for the ipsilateral foot of Vehicle group (A).

We first examined the behavioral effects of injections of the viral vectors in naïve rats. The injection of vehicle to the L4 DRG caused a slight and transient decrease of the mechanical withdrawal threshold (mechanical hyperalgesia) in the ipsilateral hindpaw that recovered after three days (Figure 2A, dashed line). Either the control vector, AdEGI, containing only the enhanced green fluorescent protein gene or the vector containing the Kir2.1 gene (AdKir) produced, on the ipsilateral (but not contralateral foot), a mild mechanical hyperalgesia that lasted for the duration of behavioral testing (14 days, Figure 2A). Thus, the presence of an adenoviral vector in the DRG even without induction of the transferred gene, produced a mild hyperalgesia on the ipsilateral foot.

In previous studies, a single injection of the inducing agent was sufficient in the rat to induce, within 24 hours, the expression of the target gene transferred by adenoviral vectors. The expression lasted for at least 10 days but could be maintained by repetitive injection of the inducing agent [7]. In the present study, we defined an induction as "immediate" or "delayed" according to whether the inducing agent was delivered, respectively, at the same time or three days after, the injection of the viral vector. The intraperitoneal injection of the inducing agent did not affect the hindpaw withdrawal threshold to mechanical stimulation in naïve, vehicle- or AdEGI-injected rats. In comparison to AdEGI, AdKir produced a similar level of mechanical hyperalgesia, regardless of whether its induction was immediate (Figure 2A) or delayed (Figure 2B).

### The mechanical hyperalgesia produced by CCD was partially alleviated by immediate, but not delayed, induction of the Kir gene

**Figure 3:**
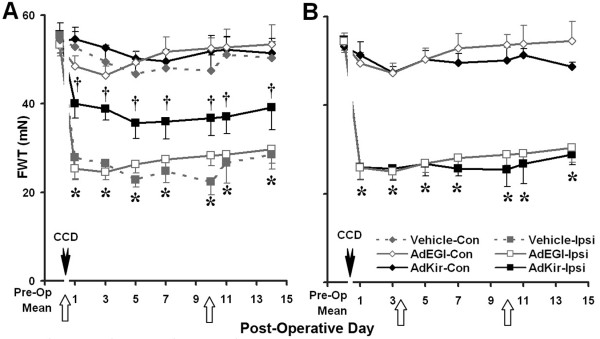
**Behavioral effects of adenoviral vectors in CCD rats**. At day 0, either the vehicle, the control vector (AdEGI), or the vector containing the Kir2.1 gene (AdKir) was delivered to the L4 DRG via sub-epineurial injection. The ganglion was then compressed (closed arrowhead). The inducing agent was delivered by IP injection (open arrow) either immediately (A) or after a delay of 3 days (B). In either case, the inducing agent was delivered again at postoperative day 10. Same format as Figure 2. Hyperalgesia (decreased mean foot withdrawal threshold, FWT) after CCD was obtained on the ipsilateral (Ipsi) but not contralateral (Con) foot. The magnitude of hyperalgesia produced by CCD (A,B, open squares, solid lines) was the same with or without the presence of the control virus (A, solid squares, dashed lines) and was greater than that produced by the control or AdKir viral vectors in the absence of CCD (Figure 2, open and closed squares). The increased hyperalgesia produced by CCD, in comparison with that produced by the viral vectors alone was significantly reduced by the induction of AdKir (A, solid squares, solid line) but only if the induction occurred at the time of compression and not if it occurred after a delay of 3 days (B). *: P < 0.05 vs. pre-operative mean for the ipsilateral foot of all groups in A-C; †: P < 0.05, AdKir vs. AdEGI or vehicle group for the ipsilateral foot (A).

CCD surgery, accompanied by the injection of either the control viral vector in vehicle or the vehicle alone, produced a long-lasting mechanical hyperalgesia on the ipsilateral foot (Figure 3A) that was indistinguishable from that obtained in response to CCD alone [4], but significantly greater than that obtained in response to either viral vector alone (Figure 2A vs. 3A, two-way ANOVA). No significant changes over preoperative values were obtained on the contralateral foot.

The injection of AdKir and its induction at the time of the CCD surgery resulted in a significantly lesser degree of hyperalgesia (higher FWTs) in the ipsilateral foot than that obtained in response to CCD with control vector or vehicle (Figure 3A). This indicated that the expression of inward-rectifying potassium channel could partially prevent the development of mechanical hyperalgesia. However, a delayed induction of AdKir failed to alleviate the hyperalgesia caused by CCD (Figure 3B).

### AdKir significantly reduced the neuronal hyperexcitability in chronically compressed DRG neurons

**Figure 4:**
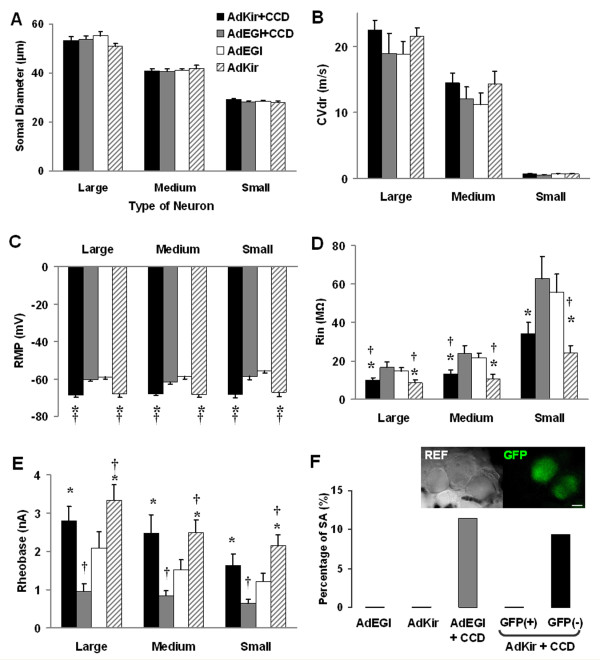
**Comparison of electrophysiological properties of DRG neurons after gene transfer and chronic compression**. Comparisons were made among the parameters of recordings obtained in vivo from DRG neurons infected with AdEGI (the control vector) or AdKir (vector expressing Kir current) in rats with or without CCD surgery. Similar effects were found within large-, medium- and small-sized DRG neurons. There was no significant difference in the somal diameter (A) and dorsal root conduction velocity (B) among different groups. Neurons infected with AdKir showed a significantly hyperpolarized resting potential (C) and lower input resistance (D) in comparison to those infected with AdEGI regardless of the presence or absence of CCD, which did not affect these two parameters. E: CCD surgery produced a significant reduction of rheobase in AdEGI-infected DRG neurons, but the effect was reversed by AdKir infection. F: Expression of Kir current (indicated by green fluorescence) also inhibited the spontaneous activity (SA) in CCD neurons. However, SA was still found in some neurons that did not express GFP in the same DRG. Inset in F: Reflected light (REF) and fluorescent (GFP) microscopic image showing the presence of viral-transferred GFP in the DRG neurons. Scale bar: 20 μm. *: P < 0.05 vs. AdEGI+CCD; †: P < 0.05 vs. AdEGI.

Under the fluorescent microscope, GFP was detected in approximately half of the neurons in the DRG injected with viral vectors (AdEGI: 52% or 66/128, AdKir: 51% or 64/126). Unless otherwise stated, all the neurons recorded were with GFP (Figure 4F inset), confirming the successful expression of target gene transferred by adenoviral vectors. In vivo intracellular recording was obtained from a total of 130 DRG neurons, including 31 neurons from AdEGI-injected rats, 28 from AdKir-injected rats, 35 from AdEGI+CCD rats and 36 from AdKir+CCD rats. The average somal diameter (Figure 4A) or dorsal root conduction velocity (Figure 4B) of neurons within each size category was not significantly different between these groups.

Among the 130 DRG neurons recorded, peripheral receptive fields were found in 60 neurons, including 35 large-, 17 medium- and 8 small-sized neurons. The large sized neurons included: 12 MSs, 16 LTMs with A-fibers (ALTMs) that were rapidly adapting (RA) to steady pressure (10 Guard-hair, 1 Down-hair and 5 glabrous), 5 slowly adapting (SA) ALTMs (3 hairy skin and 2 glabrous) and 2 mechanosensitive nociceptors with A-fibers (AMs). The medium sized neurons included: 5 MSs, 6 RA ALTMs (2 G-hair, 3 D-hair and 1 glabrous), 5 AMs and 1 AMH. The 8 small sized neurons included 4 CMs and 4 CMHs. The percentage and functional characteristics of receptive fields were not significantly different between animals in the AdEGI and AdKir, or in the control and CCD groups. The neurons with different receptive field characteristics were pooled for these groups due to the relatively small sample sizes.

The electrophysiological properties recorded from AdEGI-injected rats were not significantly different from those of vehicle-injected rats and were similar to those published for naïve rats [3,10]. CCD surgery produced neuronal hyperexcitability as indicated by a significantly lower current threshold (rheobase, reduced by approximately 50%) and the presence of spontaneous activity (in about 11.4% AdEGI-infected neurons), without any significant effect on resting membrane potential (RMP), input resistance (Rin) or dorsal root conduction velocity (CVdr) (Figure 4). These results agreed with previously published studies on the rat CCD model using the in-vitro intracellular recording techniques in our laboratory [3,5,11] and others [12,13].

**Figure 5:**
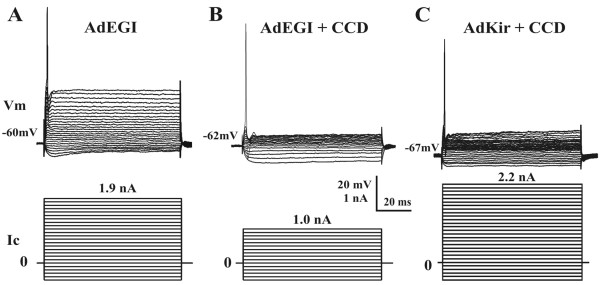
**AdKir reversed the CCD-induced hyperexcitability in DRG neurons**. Typical intracellular recordings (Vm) were obtained from the somata of DRG neurons transfected with AdEGI (A), AdEGI plus CCD surgery (B) or AdKir plus CCD surgery (C). Current steps (Ic) were injected to obtain the rheobase and input resistance. All three neurons are Aβ-type with large-diameter somata. The rheobase of DRG neurons with control vector AdEGI decreased remarkably after CCD surgery, but this effect was reversed by AdKir.

Similar effects were found within large-, medium- and small-sized DRG neurons. In comparison with neurons infected with AdEGI, and regardless of size or subjected to CCD or not, neurons infected with AdKir exhibited a significantly more hyperpolarized resting potential (Figure 4C) and a lower input resistance (Figure 4D). CCD surgery produced a significant reduction of rheobase in AdEGI-infected DRG neurons (Figure 5A and 5B), but the effect was reversed by AdKir infection (Figure 5C; summarized in Figure 4E). The expression of Kir current (indicated by green fluorescence) also inhibited the spontaneous activity (SA) in CCD neurons. CCD surgery produced SA in 11.4% of DRG neurons transfected with the control vector, AdEGI. In AdKir-injected and chronically compressed DRGs, none of the neurons expressing GFP were spontaneous active, suggesting that expression of the Kir2.1 channel inhibited the CCD-induced SA. However, SA was still found in 9.4% (3/32) of the neurons that did not express GFP in the same DRG (Figure 4F), indicating the presence of hyperexcitability in the CCD neurons not infected with adenoviral vectors.

## Discussion

We used an inducible adenoviral vector to transfer the inwardly rectifying potassium channel Kir2.1 directly into the DRG under compression. Encoded by the KCNJ2 gene, Kir2.1 is an inwardly rectifying potassium channel widely expressed in heart, blood vessels, skeleton muscles, brain as well as the Schwann cells in the peripheral nervous system [14]. Kir2.1 is believed to play a critical role in the stabilization of resting membrane potential and the modulation of cellular excitability in these tissues. A mutation of Kir2.1 contributes to a number of genetic disorders such as Andersen's syndrome [14,15]. The efficacy of the gene transfer method used in this study has been demonstrated in previous studies of neurons in the superior cervical ganglion neurons [6] and also in cardiac myocytes [7,16,17]. Our approach of sub-epineurial injection has the advantage of producing minimal side effects such as collateral tissue damage and nerve conduction block. The inducible nature of the adenoviral vector, controlled by IP injection of the inducing agent, also increases the safety of gene transfer. As shown in the electrophysiological recording experiments, AdKir significantly reduced the excitability, without altering the conduction velocity, of neurons in both control and CCD rats. Specifically, in comparison with the effects of vehicle or a control vector containing only the GFP gene, neurons infected with AdKir displayed a hyperpolarized RMP, a decreased Rin and a higher rheobase. The CCD-induced hyperexcitability, as characterized by a reduction of rheobase and increased incidence of SA, was almost totally eliminated in these AdKir-infected neurons. The development of mechanical hyperalgesia could be partially prevented if the Kir2.1 gene was induced at the beginning of CCD surgery. Taken together, our findings indicate that the expression of Kir2.1 by an inducible adenoviral vector applied locally to the DRG could reduce the neuronal hyperexcitability and mechanical hyperalgesia produced by CCD.

However, our study also revealed some limitations of this gene transfer method. First, a local injection of the adenoviral vectors (AdEGI or AdKir) produced a mild but long-lasting mechanical hyperalgesia in naïve rats. Because the injection of vehicle only caused a transient effect (hyperalgesia lasted for less than 5 days), the prolonged hyperalgesia was likely due to a local inflammation induced by the adenoviral vectors rather than the surgical procedure per se. This side effect may be resolved by using other viral vectors (e.g. adeno-associated virus) that potentially cause less tissue inflammation. Secondly, although the expression of Kir effectively reduced the neuronal excitability and eliminated the CCD-induced SA, only about half of the neurons in the DRG were successfully transfected with the target gene as indicated by GFP. In the AdKir-injected, compressed DRGs, uninfected (GFP-negative) neurons still remained hyperexcitable as indicated by an incidence of SA (9.4%) comparable to that of neurons in DRGs injected with the control-vector (11.4% SA, Figure 4). These results may partially explain the failure of relieving the hyperalgesia in the case of a delayed induction of the Kir2.1 gene. Although the electrophysiological effects were similar for neurons receiving immediate vs. delayed induction (i.e. excitability was reduced in about half of DRG neurons), a higher level of neuronal hyperexcitability in the DRG may be required to initiate than to maintain the hyperalgesia. Maintenance of chronic pain may result from mechanisms of sensitization of central neurons [18], which, once activated, are more difficult to reverse even after the initial phase even if the input from peripheral injury site is subsequently blocked [19]. Therefore the neuronal hyperexcitability contributing to chronic pain is best inhibited as soon as possible after injury.

## Conclusion

We delivered, by microinjection into the fourth lumbar DRG in rats, an inducible adenoviral vector containing a reporter gene encoding an inwardly rectifying potassium channel Kir2.1. The expression of the channel was induced either immediately or three days after the onset of a chronic compression of the DRG (CCD model of neuropathic pain). In ether case, expression of Kir2.1 significantly reduced the CCD-induced hyperexcitability of functionally characterized DRG neurons, electrophysiologically recorded in vivo. However, only the immediate and not the delayed induction of Kir2.1 significantly reduced the hyperalgesia that otherwise occurred on the hindpaw ipsilateral to the DRG. We hypothesize that a higher level of neuronal hyperexcitability in the DRG is required to initiate than to maintain the hyperalgesia and that the hyperexcitability contributing to neuropathic pain is best inhibited as soon as possible after injury.

## Methods

### Ecdysone-inducible adenovirus vector constructs

The construction of the ecdysone-inducible adenovirus vector has been described [6,7]. The viral vectors were produced in the Wilmer Gene Vector Core at The Johns Hopkins University School of Medicine (Baltimore, MD). The following three types of viral vectors were used (Figure 1A):

1) The control viral vector (AdEGI) containing only the EGFP (enhanced green fluorescent protein) gene.

2) The viral vector containing both the EGFP and the Kir2.1 gene that encodes the Kir2.1 channel (AdKir).

3) The receptor virus AdCDBEcR containing the ecdysone-inducible promoter that is subsequently mixed with AdEGI or AdKir at a ratio of 1:2 before application to the DRG. This enables the expression of target gene in the viral vectors to be controlled externally via a systemic injection of ecdysone analog [6,7].

### Surgical procedures

Adult female Sprague-Dawley rats weighing 200-250 g (n = 48 total) were housed in groups of three or four in a climate-controlled room under a 12 hr light/dark cycle. The use and handling of animals were approved by the Institutional Animal Care and Use Committee of the Yale University School of Medicine and were in accordance with guidelines provided by the National Institutes of Health and the International Association for the Study of Pain.

#### Sub-epineurial injection of viral vectors into rat DRG

Animals were deeply anesthetized with pentobarbital sodium (Nembutal, 50 mg/kg i.p.). Under a dissection microscope, the L5 transverse process was removed to expose the L4 spinal nerve. A polyethylene tube (tip diameter 100 μm) connected to a microinjection syringe was inserted into the L4 spinal nerve under the epineurium until the tip reached the center of DRG (Figure 1). The solution was slowly injected into the sub-epineurial space of DRG using a microinjection pump (5 μL in about 10 min). The adenoviral vectors AdEGI (containing EGFP gene only) or AdKir (containing both EGFP and Kir gene) were pre-mixed with the receptor virus AdCDBEcR (containing the ecdysone-inducible promoter) at a ratio of 2:1 and delivered to the DRG.

The dose of viral vectors was 10^8 ^to 10^10 ^viral particles in 5 μL of vehicle consisting of 0.7% NaCl and 5% sucrose. The vehicle containing no virus was used as a control. Briefly this two viral system works in the following way. The modified ecdysone receptor virus expresses a modified ecdysone receptor which in the presence of an ecdysone analog binds to the cell's native Retinoid X Receptor (RXR). This complex then anneals to the upstream portion (promoter) region of the ion channel virus DNA causing strong expression of the ion channel and/or the GFP reporter. The ecdysone receptor was modified in two ways one to have a stronger affinity for the native RXR and to bind the ecdysone analog, GS-E (N-(3-methoxy-2-ethylbenzoyl)-N'-(3,5-dimethylbenzoyl)-N'-tert-butylhydrazine, now known as RL1, New England Biolabs, Ipswich, MA). GS-E (45 mg in 100 μL DMSO, IP injection) was used as the inducing agent to control the time at which the expression of the transferred genes began. As previously shown, a single injection of inducing agent is sufficient to induce the expression of a target gene transferred by adenoviral vectors starting 24 hours and lasting for at least 10 days [7].

#### Rod implantation to compress the rat DRG

Immediately after sub-epineurial injection, the tube was removed and rod implantation (CCD surgery) was performed on the L4 DRG as described [1,2,4]. Briefly, the intervertebral foramen of L4 was exposed, and an L-shaped stainless steel rod (0.63 mm in diameter and 4 mm in length) was inserted to compress the L4 DRG. The incision was closed in layers. The correct placement of the implanted rod was confirmed when the ganglion was exposed for electrophysiological recording [4].

CCD surgery was performed in a total of 24 rats, including 12 injected with AdKir, 6 with AdEGI and 6 with vehicle. Another group of 24 rats, including 8 injected with AdKir, 9 with AdEGI and 6 with vehicle, did not receive rod implantation.

### Behavioral tests

Behavioral tests were administered on each of three consecutive days before and up to 14 days after viral vectors injection and rod implantation. Tests were carried out without knowledge of the type of surgery and electrophysiological results. Before testing, the rat was placed in a clear plastic cage with a metal mesh floor. After about 15 minutes of accommodation, the following tests were performed.

#### Measurement of the threshold force eliciting withdrawal to punctate indentation

A series of Von-Frey-type monofilaments each delivering a different bending force in ascending order (10, 20, 40, 60, 80, 100 and 120 mN) but having the same tip diameter of 0.1 mm were delivered to designated loci on the skin [4,10]. Each filament was applied for 1 sec, alternately to each foot and at intervals of 10 to 15 s, to each of 6 sites distributed across the plantar surface of the rat hindpaw. A Hill equation was fitted (Origin Version 6.0, Microcal Software, Inc.) to the function relating the percentage of indentations eliciting a withdrawal to the force of indentation. From this equation, the threshold force was obtained, defined as the force corresponding to a 50% withdrawal. Cutaneous hyperalgesia was defined as a postoperative decrease in threshold of 20 mN from the mean of the three preoperative thresholds.

### In-vivo intracellular electrophysiological recording

In-vivo, intracellular electrophysiological recordings were obtained from visualized neuronal somata on the surface of the DRG [20] in 18 control animals and in 20 CCD rats starting 3-5 days after injection of the inducing agent. The surgical and recording procedures were as described [3,10,20]. Briefly, under pentobarbital anesthesia (initial dose of 50 mg/kg i.p. followed by 20 mg/kg per hour as needed), the L5 transverse process was removed to expose the L4 spinal nerve, and a laminectomy was performed at the levels of L1-L6. The L4 DRG was exposed, and the L4 dorsal roots transected just prior to their entry to the spinal cord. Oxygenated ACSF was dripped periodically on to the surface of the ganglia during the surgical procedure. Under a dissecting microscope, the sheath covering the surface of the DRG (perineurium and epineurium) was carefully removed using fine forceps and scissors. Animal was transferred to a platform attached to the recording table. The spinal processes were clamped at T12 and S1 region, and a skin pool was formed by suturing to a metal ring. The ganglion was then placed on a spoon-shaped platform under the microscope (BX50WI, Olympus Optical, Tokyo, Japan). The platform holding the ganglion and the animal were mechanically isolated from each other allowing the lower limb to be manipulated during the search for receptive fields without transmitting unwanted mechanical stimuli to the recorded cell. The DRG was continuously perfused at a rate of 3~4 ml/min with oxygenated artificial cerebrospinal fluid (ACSF). The ACSF contained (in mM): 130 NaCl, 3.5 KCl, 24 NaHCO_3_, 1.25 NaH_2_PO4, 1.2 MgCl_2_, 1.2 CaCl_2_, and 10 Dextrose. The solution was bubbled with 95% O_2 _and 5% CO_2 _and had a pH of 7.4 and an osmolarity of 290~310 mOsm. The temperature of the ACSF around the ganglion was maintained at about 35°C by a heater and controller (TC-344A, Warner Instrument, Hamden, CT). The intracellular recording electrodes, fabricated from borosilicate glass (World Precision Instruments, Sarasota, FL), were pulled on a Flaming/Brown micropipette puller (P-97, Sutter Instrument, Novato, CA) and filled with 1.0 M KCl (impedance: 40~80 MΩ). Electrical pulses were delivered through a suction electrode applied at the cut end of the dorsal root.

Electrophysiological recordings were collected with bridge mode under current clamp using an AxoClamp-2B (Molecular Devices, Palo Alto, CA), stored digitally via a Digidata 1322A interface, and analyzed offline with pClamp 8 software (Molecular Devices). The electrode resistance was balanced by a bridge circuit in the amplifier. A neuron was accepted for study only when it exhibited a resting membrane potential (RMP) more negative than -45 mV. Current steps, each 100 ms duration, were delivered through the intracellular recording electrode in increments of 0.05 nA from -0.5 to 4 nA. The input resistance (Rin, MΩ) was calculated from the slope of a steady-state I-V plot obtained from responses to a series of hyperpolarizing currents steps from -0.5 to -0.05 nA. The current threshold (Rheobase, nA) was defined as the minimal depolarizing current required to evoke an action potential.

### Classification of neurons

The neurons on surface of the DRG were viewed with reflected-light epi-illumination or by epifluorescence [20]. The fluorescent mode enabled the confirmation of the presence of GFP and thereby the expression of target gene (Figure 5D inset). Using the reflected-light mode of viewing, a neuron to be recorded was initially selected according to the size of its soma and, based on the mean of its longest and shortest diameter, categorized as small (S, < = 30 μm), medium (M, 31~45 μm) or large (L, >45 μm) [5,10]. A dorsal-root axon was classified as a C-, Aδ-, or Aα/β- fiber according to whether its respective dorsal root conduction velocity (CVdr) was < 1.2 m/s, < 7.5 or >7.5 m/s [5,11,21].

Each neuron was further classified by its receptive field properties using a limited set of mechanically delivered search stimuli. Aside from gentle extension/flexion of the toes or angle used to identify neurons with muscle spindles, ("MS"). the search stimuli were applied primarily to the skin of the foot and lower leg. A cutaneous neuron was initially broadly categorized as either "low-threshold" mechanosensitive (LTM) if it responded readily to gentle pressure or stroking of the skin with the experimenter's finger or "nociceptive" if it could only be activated by mildly noxious stimuli such as pinching of the skin (experimenter's thumb and forefinger). A glass probe was used to outline the borders of the receptive field.

Nociceptive neurons were further classified by whether they responded to noxious heat or cold stimuli. Heat stimuli were delivered by a contact thermode consisting of a chip resistor (2 × 3 mm^2^) that delivered precise control over temperatures the skin/thermode interface. Each heat stimulus was a ramp (20°C/sec) from a controlled base temperature of 38°C to a plateau (51°C) which was maintained for 5 sec followed by passive cooling back to base. The cold stimulus was produced by an ice chip that was placed on the skin for 20 sec. Nociceptive neurons responsive to noxious mechanical, heat and cold with A- or C-fibers were termed A- or CMH and, if responsive to cold, A- or CMHCs.

### Statistical Analyses

Data values are presented as means with standard errors of the mean. Statistical analysis was performed using SigmaStat (Version 2.03, SPSS Inc. San Rafael, CA) computer software. Student's t-tests were used to determine the statistical significance of differences between means obtained from two different groups of neurons. One- or two-way analyses of variance (ANOVAs) followed by post hoc pairwise comparisons (Student-Newman-Keuls method) were used to determine the statistical significance of differences between means obtained from three or more experimental groups. For a given foot, the mean threshold forces to elicit foot withdrawal to punctuate indentation obtained for each of the four surgical groups were analyzed with a two-way ANOVA (group × days) with repeated measures on days. Post hoc contrasts determined the significance of differences between the mean of the three preoperative tests and the mean obtained for each postoperative test. Chi-Square tests were used to assess differences in the percentages of neurons with SA. A probability of 0.05 was chosen as the criterion for significance.

## Competing interests

The authors declare that they have no competing interests.

## Authors' contributions

CM, RHL and DCJ designed the experiments, DCJ and JR produced the viral vector. CM collected and analyzed the data and wrote the manuscript with contributions from RHL. All authors read and approved the final manuscript.
